# Metabolic Plasticity of Cancer Stem Cells in Response to Microenvironmental Cues

**DOI:** 10.3390/cancers14215345

**Published:** 2022-10-29

**Authors:** Yunong Xie, Stephanie Ma, Man Tong

**Affiliations:** 1 School of Biomedical Sciences, The Chinese University of Hong Kong, Hong Kong 999077, China; 2 School of Biomedical Sciences, Li Ka Shing Faculty of Medicine, The University of Hong Kong, Hong Kong 999077, China; 3 State Key Laboratory of Liver Research, The University of Hong Kong, Hong Kong 999077, China; 4 The University of Hong Kong—Shenzhen Hospital, Shenzhen 518000, China

**Keywords:** cancer stem cells, cancer metabolism, tumor microenvironment, plasticity

## Abstract

**Simple Summary:**

Accumulating evidence indicates the existence of cancer stem cells (CSCs) sub-populations which fuel cancer growth and maintain stemness in different cancers. In addition to the genetic and phenotypic variabilities that differentiate CSCs from non-CSCs counterparts, CSCs adopt a flexible metabolic strategy to sustain their oncogenic and stemness properties, in order to survive and propagate in a hostile tumor microenvironment (TME). TME factors and metabolites exert context-dependent influence on cancer stemness. In addition, the metabolic landscape in TME is complicated by the crosstalk between CSCs and tumor-infiltrating cells. In this review, we will summarize the metabolic interaction between CSCs and various microenvironmental factors and review how this interplay regulates cancer stemness and tumorigenesis.

**Abstract:**

An increasing body of evidence suggests that cancer stem cells (CSCs) utilize reprogrammed metabolic strategies to adapt to a hostile tumor microenvironment (TME) for survival and stemness maintenance. Such a metabolic alteration in CSCs is facilitated by microenvironmental cues including metabolites such as glucose, amino acids and lipids, and environmental properties such as hypoxic and acidic TME. Similarly, metabolites uptake from the diet exerts critical imprints to the metabolism profile of CSCs and directly influence the maintenance of the CSC population. Moreover, CSCs interact with tumor-infiltrating cells inside the CSC niche to promote cancer stemness, ultimately contributing to tumor development and progression. Understanding the underlying mechanisms of how CSCs employ metabolic plasticity in response to different microenvironmental cues represents a therapeutic opportunity for better cancer treatment.

## 1. Introduction

Emerging evidence has suggested that tumor cells inside a tumor bulk are heterogenous with variable genetic, phenotypic and functional profiles [1]. Among which, subpopulations of tumor cells with enhanced tumor-initiating and self-renewal abilities, termed cancer stem cells (CSCs), have been identified and characterized in multiple cancer types [2,3,4,5,6,7]. Growing studies have reported compelling evidence that CSCs are the root of tumor initiation [8], progression [9], relapse [10], and metastasis [11], which pinpoint CSCs to be a potential therapeutic target for curative cancer treatment.

CSCs reside in the tumor microenvironment (TME) where heterogeneous cell populations interact with one another [12]. The interaction through the secretion and uptake of various types of molecules results in either promotion or suppression of tumor development and progression [13]. Metabolites have been recognized as one of the critical signaling molecules that facilitate the metabolic interaction among different cells in the TME [14].

Reprogramming cellular metabolism is considered as one of the core hallmarks of cancer [15,16], by which cancer cells harness the advantage of metabolic adaptations to produce energy or new biomass and sustain uncontrolled proliferation and viability [17,18]. CSCs, like most cancer cells, are able to reprogram their metabolism to better adapt to the environmental changes [19]. However, how CSCs respond differently compared with their non-CSCs counterparts under different microenvironmental cues has not been systemically reviewed. In light of this, the metabolic plasticity of CSCs in response to different microenvironment factors will be summarized.

## 2. Metabolites as Signaling Messengers between CSCs and TME

Extracellular metabolites could exert either tumor-promotive or -suppressive effect in different cancers (Table 1). Their availabilities could direct the intracellular signaling pathways for the regulation of tumorigenesis and cancer stemness.

### 2.1. Availabilities of Metabolites in the TME Direct CSC Properties

Glucose, as the primary energy-producing metabolite, has been shown in different cancers to either promote or suppress CSCs. A study observed that hyperglycaemia resulted in enhanced invasion and stemness of breast CSCs by reducing the tumor suppressing microRNA miR-424 [20]. However, other studies suggested that CSCs might also expand their population and maintain their stemness under restricted glucose availability in the TME. CD44^+^CD117^+^ CSCs isolated from epithelial ovarian cancer patients showed increased glucose uptake while CSC phenotype could still be maintained under glucose deprivation environment [21]. Similarly, a recent study conducted by our group found that glucose deprivation could promoted cancer stemness and drug resistance, concomitant with an increase population of CD133^+^ liver CSCs [22]. These studies indicate that extracellular glucose is a key regulator for cancer stemness, but its effect is context-dependent.

Amino acids are another important group of metabolites which influence CSC stemness [42]. Glutamine is an abundant amino acid fueling the tricarboxylic acid (TCA) cycle and supporting the biosynthesis of other metabolites [43]. Although glutamine dependence in cancer cells has been extensively investigated [44], limited studies have explored the role of glutamine in CSCs until recent years. Preferential up-regulation of glutamine transporter SNAT2 in gastric CSCs led to an increased glutamine uptake and thereby promoted cancer stemness in gastric cancer [23]. In ovarian cancer, deprivation of glutamine promoted cancer stemness possibly through mitochondrial fragmentation resulted from increased phosphorylation of mitochondrial regulator DRP1 [24]. In liver cancer, fresh patient tumor samples with more necrotic areas had lower glutamine concentration and a higher OCT4 expression [25]. Primary glioblastoma cultures with CD133 expression showed a reduced glutamine uptake and utilization and displayed a more mesenchymal-like signature compared with CD133 negative counterparts [26]. In addition to glutamine, other amino acids were shown to regulate cancer stemness. Baksh et al., reported that reducing extracellular serine level led to the differentiation of epidermal stem cells which were shown to be the origin of squamous cell carcinoma [28]. Terasaki et al., reported the stemness-promotive role of glycine which could support epithelial-mesenchymal transition in colorectal CSCs [29]. Leukemic stem cells (LSCs) isolated from primary human acute myeloid leukemia (AML) specimens with low reactive oxygen species (ROS) levels were more dependent on the uptake and catabolism of amino acids to fuel oxidative phosphorylation (OXPHOS) [30].

Extracellular lactate impacts cancer cells and CSCs through diverse mechanisms [31,32,33,34]. In glioma CSCs which rely on OXPHOS for energy production, lactate serves as an energy source and induces metabolic rewiring of CSCs to maintain an aggressive phenotype [31]. In oral squamous cell carcinoma (OSCC), extracellular lactate activated Wnt signaling and increased CSC marker (CD133) expression in organoids generated from fresh OSCC specimens, while inhibiting the transport of lactate into the cells abrogated the CSC phenotypes [32]. In liver CSCs, exogenous lactate treatment enhanced lactylation on the lysine residues of histone H3 and promoted hepatic tumorigenicity [33]. Reducing lactate production through inhibition of lactate dehydrogenase significantly impaired CSC tumor-initiating function in lung cancer [34].

In addition to glucose, amino acids and lactate, lipids also play critical roles in regulating the stemness trait of CSCs. In ovarian CSCs, the provision of exogenous fatty acid sources such as palmitoleic acid and oleic acid rescued CSCs from ferroptosis-induced cell death which was resulted from the inhibition of fatty acid lipogenesis enzyme stearoyl-CoA desaturase (SCD1) [35]. In relapsed/refractory AML patients, relapsed LSCs isolated from patient samples with low ROS levels could compensate for the depletion of amino acid metabolism through increased uptake of fatty acids to support energy metabolism [30,36]. Non-adherent mammosphere culture established from patients with breast-to-brain metastasis showed increased self-renewal and proliferation when supplemented with palmitic acid [37].

### 2.2. CSC Metabolite Secretome Affects Tumor Initiation and Progression

CSCs consume metabolites in the TME, at the same time CSCs also actively remodel the TME by their altered metabolite secretome in order to establish a supportive ecosystem for tumor initiation and progression [45]. Under hypoxic condition in glioblastoma, adenosine production was significantly increased via a positive feedback loop mediated by prostatic acid phosphatase and adenosine receptor A2B (AB2R), resulting in an enhanced proliferation of glioblastoma CSCs [38]. Similarly, Niechi et al., observed that increased adenosine production facilitated the adhesion, migration, and invasion abilities of glioblastoma CSCs [46]. In non-small cell lung cancer (NSCLC) CSCs, increased CD73/adenosine pathway promoted the resorption of osteoclasts in a co-culture system, leading to the metastasis of NSCLC CSCs into the bone [39]. Moreover, adenosine has been acknowledged as an immunosuppressive metabolite [47,48,49], suggesting its role in promoting immune evasion of cancer cells.

Ketone bodies, comprising of acetone, acetoacetate and beta-hydroxybutyrate (β-HB), possess both pro-tumor and anti-tumor properties [50]. Increased ketone body production was first observed in normal intestinal stem cells [51]. Increased β-HB promoted self-renewal of intestinal stem cells by activating Wnt-related signaling [51]. Using engineered ketone body-producing fibroblasts, Martinez-Outschoorn et al., showed that increased ketone body production enhanced tumor growth and metastasis of breast cancer through the activation of ketone body metabolic enzymes OXCT1/2 and ACAT1/2 [40]. Similarly, the same research group discovered that adding ketone bodies in the cell culture of MCF7 human breast cancer cells significantly enhanced the stemness-related gene signatures [52]. However, other evidence indicates that ketone bodies could exert a suppressive effect on tumor progression. Our group previously reported that suppressed ketone body production resulted from deranged tyrosine catabolism activated mTOR signaling and promoted early hepatic tumorigenesis, indicating an anti-tumor effect of ketone bodies in hepatocellular carcinoma (HCC) [41]. Moreover, Dmitrieva-Posocco et al., discovered a tumor-suppressive effect of β-HB which activates an anti-tumor signaling cascade mediated by Hcar2 and Hopx in colorectal cancer [53].

## 3. Microenvironmental Cues Reprogram CSC Metabolism

**Figure 1:**
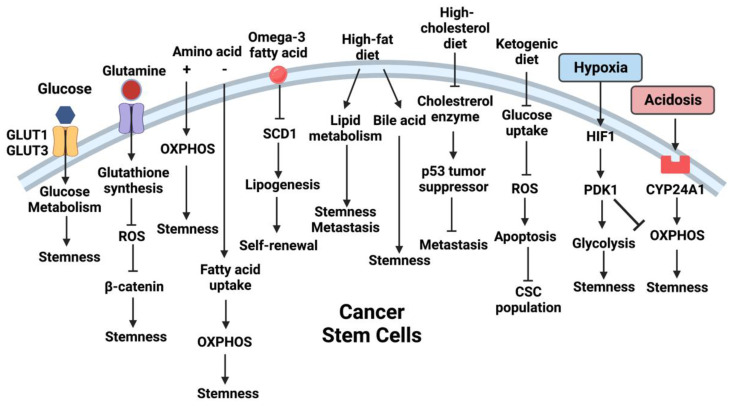
TME and extracellular metabolites reprogram CSC metabolism. Schematic diagram illustrating the effects of TME metabolites, diet and environmental factors on regulating the metabolism and functions of CSCs.

Metabolic plasticity could be observed in CSCs exhibiting flexible energy metabolic strategy, that enables them to cope with energy demands in response to changing nutrient availability and environmental stress (Figure 1) [54,55,56]. For instance, glioblastoma stem cells derived from glioblastoma cell line U87 were shown to embrace a glycolytic metabolism under hypoxic condition as evidenced by reduced mitochondrial respiration and enhanced glycolysis [57]. On the other hand, two other independent studies reported that patient-derived glioblastoma stem cells harness OXPHOS to support their CSC properties [58,59]. It is interesting to note that the preference of energy metabolic pathway is affected by the experimental models. Enhanced glycolysis was observed in CSCs derived from in vitro cell line models [57,60,61], whereas OXPHOS supported CSCs isolated from spontaneous tumor models and fresh patient samples [21,58,62]. A possible explanation is that in vitro cell culture models usually fail to recapitulate the complexity of metabolite availability in the TME [63]. Moreover, other TME factors such as limited oxygen availability (hypoxia) and extracellular acidity also contributes to the regulation of cancer stemness [64,65]. A comprehensive understanding of the metabolic adaptations of CSCs is warranted to shed light on the development of novel therapeutics.

### 3.1. Alterations in TME Reprogram CSC Metabolism

Under hypoxia, CSCs rely on glycolysis rather than OXPHOS to maintain their survival [56]. Zhou et al., observed that glioblastoma stem cells maintained stemness properties in hypoxic condition through enhanced glycolysis and suppressed mitochondrial respiration [57]. Lan et al., reported that hypoxia induced the expression of AB2R which further regulated interleukin-6 and NANOG expression to promote breast CSC enrichment [66]. An independent study in breast cancer also showed that hypoxia promoted breast CSC properties through pyruvate dehydrogenase kinase 1 (PDK1) [67]. In HCC, hypoxia promoted tumorigenesis and cancer stemness through stabilizing and activating hypoxia-inducible factor 1 alpha (HIF-1α) [68].

Acidic extracellular microenvironment, termed acidosis, is another physical environmental factor that influences CSC metabolism and functions [65,69,70,71]. In melanoma CSCs, acidic extracellular microenvironment helped sustain oxidative metabolism by up-regulating the stemness marker SOX2 [65]. In glioma stem cells, acidic stress rewired energy metabolism to favor mitochondrial respiration through CYP24A1/vitamin D regulation [72]. Moreover, in colorectal CSCs, acidosis suppressed vitamin metabolism by inhibiting vitamin D receptor expression, thus promoting CSC functions [73].

### 3.2. Metabolites in TME Reprogram CSC Metabolism

In addition to the alteration of physical properties of the TME, extracellular metabolites also contribute to the reprogramming of CSC metabolism. High glucose concentrations in the culture media promoted glucose metabolism in pancreatic and ovarian CSCs as evident by the activation of glucose transporter 1 (GLUT1) [74]. In another study conducted by Flavahan et al., reduced glucose concentration in the culture media (2.5 mM) promoted survival and growth of brain tumor-initiating cells through glucose transporter type 3 dependent glucose uptake [75]. Glutamine, as another important nutrient, has also been associated with the maintenance of cancer stemness. Liao et al., found that glutamine promoted glutathione synthesis and suppressed excessive intracellular ROS, which in turn supported the maintenance of cancer stemness through β-catenin signaling pathway in stem-like side population (SP) cells from lung and pancreatic cancers [27]. Fatty acids have also been implicated in regulating cancer stemness. In breast cancer, extracellular omega-3 polyunsaturated fatty acids inhibited SCD1-mediated lipogenesis which suppressed self-renewal and tumor-initiating abilities of breast CSCs [76].

The reprogrammed metabolic strategies in CSCs represent therapeutic opportunities. Drugs targeting glucose metabolism exert effect on glycolytic CSCs, as exemplified by the use of 2-DG [77,78,79]. On the other hand, targeting the mitochondrial respiratory machinery by OXPHOS inhibitor metformin showed therapeutic effects in clinical trials of colorectal adenoma [80]. These studies indicate that understanding CSC metabolic plasticity and metabolic vulnerability could direct the development of more effective cancer therapy.

## 4. Dietary Effects on CSC Metabolism

Diet determines the nutrient availability in the microenvironment where CSCs are exposed to, that in turn alters CSC metabolism [81].

High fat diet has been correlated with higher cancer incidence and worse prognosis [82], and it has recently been linked with the maintenance of stemness in both normal stem cells [83] and CSCs [37,84,85,86]. In normal intestinal stem cells, Beyaz et al., identified that high fat diet induced the expansion of Lgr5^+^ intestinal stem cell pool through the activation of peroxisome proliferator-activated receptor-related signaling pathway [83]. In oral carcinoma CSCs, high fat diet was found to enhance lipid metabolism by increasing the expression of fatty acid receptor CD36 and lipid metabolic genes, which contribute to increased metastasis of CSCs [84]. In high fat diet-induced breast cancer, Wang et al., discovered that fatty acid β-oxidation activated through Jak/STAT3 signaling promoted self-renewal and chemoresistance properties of breast CSCs [37]. Similarly, high fat diet expanded the LGR5^+^ CSCs pool and promoted tumorigenesis through JAK2/STAT3 signaling in colon cancer [85]. Moreover, high fat diet resulted in the accumulation of bile acids including tauro-β-muricholic acid and deoxycholic acid, which antagonized intestinal FXR and promoted proliferation of LGR5^+^ CSCs [86].

Increased dietary intake of cholesterol and high cholesterol level have been implicated to facilitate tumor development and progression [50]. Ehmsen et al., observed that cholesterol biosynthesis-related proteins were up-regulated in breast CSCs, while inhibiting cholesterol synthesis impaired CSC properties [87]. Similarly, Wang et al., found that increased cholesterol biosynthesis and excessive cholesterol intake from diet contributed to enhanced proliferation, stemness and tumorigenic properties of intestinal stem cells [88]. Mechanistically, excessive dietary cholesterol reduced the level of cholesterol enzyme squalene epoxidase, which in turn suppressed the GSK3β/p53 tumor suppressive pathway and promoted the progression and metastasis in colorectal cancer [89]. These studies indicate a critical role of cholesterol in sustaining the CSC populations.

Other types of diets have also been implicated in regulating CSCs. For example, a fasting-mimicking diet was observed to lower the glucose level and reduce the expression of stemness markers in breast CSCs [90]. Accumulating evidence has attributed the anti-tumor effect of ketogenic diet to its influence on CSC properties. Ketogenic diet has been shown to benefit cancer treatment through reprogramming cancer cell metabolism [91,92]. Ji et al., observed that co-culturing glioblastoma CSCs with β-HB, a ketone body produced under ketogenic diet, inhibited glucose uptake and increased ROS production, resulting in apoptosis of CSCs [93].

Results from the above studies indicate that dietary metabolites possess either promotive or suppressive role in the maintenance of CSC phenotypes in various types of cancers. Deciphering the mechanisms of such dietary effects on CSCs would shed light on novel therapeutic opportunities.

## 5. Metabolic Interaction of Tumor-Associated Cells in TME

**Figure 2:**
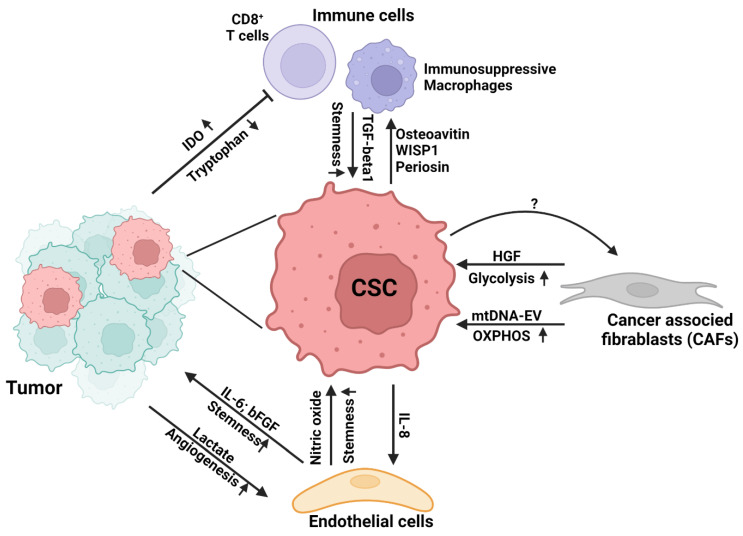
Cancer-immuno-metabolic crosstalk. Schematic diagram illustrating the bi-directional interaction of tumor cells including CSCs with the neighboring non-tumor cells through TME factors.

The presence of heterogeneous cell populations within the tumor bulk are well characterized in different cancers [18,94]. CSCs, non-CSC counterparts, cancer-associated fibroblasts, endothelial cells, immune cells, and other cell populations interact with each other and contribute to the regulation of tumor development and progression [95] (Figure 2). Their interactions in the tumor niche have been extensively reviewed previously, with the focus on their crosstalk through signaling molecules such as cytokines and chemokines [96]. In particular, inflammatory molecules have been shown to impact CSC phenotypes [97,98]. However, the metabolic connection between CSCs and neighboring cells in TME has not been systematically reviewed.

### 5.1. Cancer Associated Fibroblasts

Cancer associated fibroblasts (CAFs) are fibroblastic cells residing in the TME that possess regulatory functions on cancer cells and CSCs [99]. CAFs function to reprogram metabolism of neighboring cancer cells and CSCs through the secretion of various signaling molecules [100]. Yan et al., found that hepatocyte growth factor secreted by pancreatic CAFs triggered the stemness potential and enhanced glycolysis through the activation of YAP/HIF-1α signaling in pancreatic cancer [101]. Moreover, CAFs are known to trigger a phenomenon termed the “reversed Warburg effect” [102], a process where glycolytic, lactate-producing CAFs coupled with the OXPHOS-dependent cancer cells to promote tumorigenesis [103]. CAFs isolated from prostate hyperplasia employed glycolytic metabolism as evident by an increased lactate production and export [104]. Lactate produced from CAFs in turn was taken up by prostate cancer cells after co-culture to support proliferation [105]. A direct evidence of CAFs-CSCs metabolic interaction was observed in breast cancer, where Pasquale et al., found that the whole genome mitochondrial DNA (mtDNA)-loaded extracellular vehicles (EVs) secreted from CAFs could be transferred into breast CSCs and thus restored OXPHOS metabolism [105]. However, mutual metabolic influence between CSCs and CAFs has not been thoroughly studied and may represent a research opportunity, considering high OXPHOS dependency in certain types of CSCs.

### 5.2. Endothelial Cells

Endothelial cells (ECs) are functional cells that line the vascular system and are recruited to the TME for angiogenesis [106,107]. Unlike other non-malignant cells, anaerobic glycolysis is the primary metabolic pathway for energy production in ECs, with lactate as the major end product [108,109,110,111]. ECs can also import the microenvironmental lactate for ATP production [112,113]. In colorectal and breast cancers, Vegran et al., reported that the excessive lactate secreted by tumor cells facilitated the angiogenesis function of ECs through the activation of NFkB/interleukin 8 (IL-8) pathway [112], indicating a direct metabolic interaction between tumor cells and tumor-associated ECs. The interaction between ECs and CSCs was previously reported by Krishnamurthy et al., where they showed that interleukin-6 secreted by ECs promoted CSC phenotypes through the activation of STAT3 signaling [114]. Our group has previously reported that enhanced secretion of proinflammatory cytokine IL-8 from CD133^+^ liver CSCs induced tumor angiogenesis [115]. Wang et al., observed that co-culturing of colorectal cancer cells with the conditioned medium of ECs increased the expression of NANOG and expanded the CSC population [116]. Similarly, Fessler et al., identified that basic fibroblast growth factor secreted by tumor microvascular ECs was responsible for the induction of cancer stemness phenotype in glioblastoma [117]. In glioma, nitric oxide present in the perivascular niche promoted neurosphere-forming and tumorigenic capacities through the activation of NOTCH signaling [118]. Despite the current findings, more direct evidence to show the interaction between ECs and CSCs in the TME are warranted.

### 5.3. Immune Cells

Immune evasion is one of the major hallmarks of CSCs [119]. The metabolic crosstalk between tumor-infiltrating immune cells and cancer cells has been well recognized. The scarcity of glucose and oxygen in the TME as a result of enhanced glycolysis in cancer cells facilitated the metabolic reprogramming of immune cells [120]. Zhang et al., observed that under hypoglycemia and hypoxic conditions, CD8^+^ tumor-infiltrating T cells shifted their metabolism from glycolysis towards fatty acid catabolism [121]. Furthermore, colorectal cancer cells facilitated immune evasion through the expression of indolamine 2, 3-dioxygenase, an enzyme that reduces the tryptophan availability to the tumor-infiltrating T cells [122]. The interaction between CSCs and immune cells through immune regulatory molecules including cytokines, chemokines and immune checkpoints has been widely studied [123]. Proteins secreted from CSCs such as osteoactivin, Wnt-induced signaling protein 1 and periostin were identified to recruit tumor-supportive macrophages, resulting in immune evasion [124,125,126]. Immune cells also secreted signaling molecules such as transforming growth factor- beta1 to maintain CSC properties [127]. However, the direct metabolic crosstalk between CSCs and immune cells has yet to be investigated. Further studies will be required to reveal the metabolic interplay between CSCs and immune cells, so as to devise novel therapeutic strategies to overcome immune evasion.

## 6. Conclusions and Future Perspectives

The TME is comprised of various cellular and non-cellular components which reside with CSCs in the tumor bulk and continuously affect and modify the behaviors of CSCs. Metabolic plasticity displayed by CSCs enables flexible switching of their metabolic strategies to accommodate and survive in the hostile TME. In reverse, CSCs produce and secrete various proteins and metabolites to influence the neighboring microenvironment. This bidirectional interaction not only sustains the aggressive tumor behaviors but also protects and enhances tumor survival in response to stress and environmental insults.

Given the importance of metabolic reprogramming in the maintenance of cancer stemness, elucidating the underlying molecular mechanisms which drive the metabolic plasticity of CSCs will likely reveal novel metabolic vulnerabilities and therapeutic targets to combat CSC-driven tumor development and progression. Current research efforts have been put to identify activated or mutated metabolic enzymes which play roles in promoting certain metabolic pathways to support cancer development and progression. Clinical trials with the use of small molecular inhibitors to target different metabolic enzymes has been underway [128]. Metformin is an example of metabolic drugs that have been shown to target CSC signaling pathways and CSC metabolism in preclinical studies [129,130]. Dietary interventions, such as caloric restriction diet, fasting diet, ketogenic diet and dietary supplements used alone or in combination with other molecular inhibitors, are also attractive approaches to target cancer and CSC metabolism [131].

Despite the enthusiasm of the development of metabolic therapies to target CSC metabolism for cancer treatment, concern and criticism were raised regarding the validity of experimental results using in vitro culture which deviates from the physiological environment [132]. In particular, most of the past studies investigating CSC functions and metabolism involved the culture of CSCs in an in vitro setting. Therefore, further studies with the use of cancer models which highly mimic physiological conditions are still required to unveil the metabolic vulnerabilities of CSCs for new drug development.

## Figures and Tables

**Table 1:** The effect and regulation of extracellular metabolites on cancer stemness in different cancers.

Metabolite	Effect	Cancer Type	Mechanism
Glucose	Promotive [20]	Breast cancer	[+]Glucose → MicroRNA miR424 activity ↓ → Tumorigenesis & cell invasion ↑
	Promotive [21]	Ovarian cancer	[+]Glucose → OXPHOS ↑ → CSC phenotype [−]Glucose → Maintenance of CSC phenotype in CD44^+^CD117^+^ CSCs
	Inhibitory [22]	Hepatocellular carcinoma	[−]Glucose → FUT1 expression ↑ → Stemness ↑
Glutamine	Promotive [23]	Gastric cancer	[+]Glutamine → Glutamine transporter SNAT2 ↑ → Stemness ↑
	Inhibitory [24]	Ovarian cancer	[−]Glutamine → ROS ↑ → MAPK/ERK ↑ → DPR1 phosphorylation ↑ → Stemness ↑
	Inhibitory [25]	Hepatocellular carcinoma	[−]Glutamine → Rictor/mTORC2 ↑ → Stemness ↑
	Inhibitory [26]	Glioblastoma	[−]Glutamine → CD133^+^ CSC population ↑
	Promotive [27]	Non-small cell lung cancer	[+]Glutamine → ROS ↓ → β-catenin↑ → Stemness ↑
Amino acids	Promotive (Serine) [28]	Oncogenic epidermal stem cells	[+]Serine → De novo serine synthesis ↓ → α-ketoglutarate production ↓ → Repressive histone modification H3K27me3 ↑ → Stemness ↑
	Promotive (Glycine) [29]	Colorectal cancer	[+]Glycine → Wnt signaling → Stemness ↑
	Promotive [30]	Acute myeloid leukemia	[+]Amino acids → ROS ↓ → CSC population ↑
Lactate	Promotive [31]	Glioma	[+]Lactate → OXPHOS ↑ → Aggressiveness and stemness ↑
	Promotive [32]	Oral squamous cell carcinoma	[+]Lactate → Epithelial-mesenchymal transition↑ → Stemness ↑
	Promotive [33]	Hepatocellular carcinoma	[+]Lactate → H3 histone lactylation → Tumorigenesis ↑
	Promotive [34]	Lung cancer	[+]Lactate → Pyruvate metabolism reprogramming → Cell proliferation and survival
Fatty acid	Promotive (Palmitoleic/oleic fatty acid) [35]	Ovarian cancer	[+]Palmitoleic/oleic acid → Ferroptosis ↓ → CSC population ↑
	Promotive [30,36]	Acute myeloid leukemia	[+]Fatty acid uptake → Energy metabolism ↑ → Maintenance of CSC population
	Promotive (Palmitic acid) [37]	Breast cancer	[+]Palmitic acid → Self-renewal and proliferation ↑
Adenosine	Promotive [38]	Glioblastoma	[+]Adenosine → Aggressive CSC phenotype ↑
	Promotive [39]	Lung cancer	[+]Adenosine → Metastasis
Ketone body	Promotive [40]	Breast cancer	[+]Ketone body → OXCT1/2↑ → Cancer stemness ↑
	Suppressive [41]	Hepatocellular carcinoma	[−]Ketone (β-HB) → mTOR pathway → Tumorigenesis

↑ Increase/Activation; ↓ Decrease/Suppression; + Increase in abundance; − Decrease in abundance; → Cause.
